# Supplementary material for: The effect of pre-resection obesity on post-resection body composition after 75% small bowel resection in rats
Source: Sci Rep. 2021 Jun 21;11:13009. doi: 10.1038/s41598-021-92510-7 (PMC8217239; doi:10.1038/s41598-021-92510-7)
Supplement: Supplementary file 4 — Supplementary Information 4. [file 41598_2021_92510_MOESM4_ESM.pdf]

Title Page/Supplementary File

Title:
"The effect of pre-resection obesity on post-resection body composition after 75% small bowel resection in rats"

Author List:
Neesha S. Patel, Ujwal R. Yanala, Shruthishree Aravind, Roger D. Reidelberger, Jon S. Thompson, Mark A. Carlson

Contents:

Fig. S1	pdf file
Fig. S2	pdf file
Fig. S3	pdf file
Fig. S4	pdf file
Fig. S5	pdf file
Fig. S6	pdf file
Fig. S7	pdf file
Fig. S8	Excel file
Fig. S9	Excel file
Fig. S10	pdf file
